# Modeling maize growth and nitrogen dynamics using CERES-Maize (DSSAT) under diverse nitrogen management options in a conservation agriculture-based maize-wheat system

**DOI:** 10.1038/s41598-024-61976-6

**Published:** 2024-05-23

**Authors:** Kamlesh Kumar, C. M. Parihar, H. S. Nayak, D. R. Sena, Samarth Godara, Rajkumar Dhakar, Kiranmoy Patra, Ayan Sarkar, Sneha Bharadwaj, Prakash Chand Ghasal, A. L.Meena, K. Srikanth Reddy, T. K. Das, S. L. Jat, D. K. Sharma, Y. S. Saharawat, Upendra Singh, M. L. Jat, M. K. Gathala

**Affiliations:** 1 ICAR-Indian Agricultural Research Institute (IARI), New Delhi, India; 2 ICAR-Indian Institute of Farming System Research, Modipuram, Meerut, U.P. India; 3 Cornell University, Ithaca, NY USA; 4 International Water Management Institute, New Delhi, India; 5 ICAR-Indian Agricultural Statistical Research Institute (IASRI), New Delhi, India; 6 ICAR-Indian Agricultural Research Institute (IARI), Gogamukh, Assam India; 7 ICAR-Indian Institute of Maize Research (IIMR) Unit Delhi, New Delhi, India; 8 International Fertilizer Development Centre IN (Center US), Alabama, USA; 9 International Fertilizer Development Centre- Alabama US (International Center), Muscle Shoals, USA; 10 International Crops Research Institute for the Semi-Arid Tropics (ICRISAT), Patancheru, Hyderabad, India; 11 International Maize and Wheat Improvement Center (CIMMYT), South Asia Regional Office, Dhaka, Bangladesh

**Keywords:** Ammonia volatilization, CERES-Maize, DSSAT, Nitrate leaching, Zero tillage, Plant sciences, Environmental sciences

## Abstract

Agricultural field experiments are costly and time-consuming, and often struggling to capture spatial and temporal variability. Mechanistic crop growth models offer a solution to understand intricate crop-soil-weather system, aiding farm-level management decisions throughout the growing season. The objective of this study was to calibrate and the Crop Environment Resource Synthesis CERES-Maize (DSSAT v 4.8) model to simulate crop growth, yield, and nitrogen dynamics in a long-term conservation agriculture (CA) based maize system. The model was also used to investigate the relationship between, temperature, nitrate and ammoniacal concentration in soil, and nitrogen uptake by the crop. Additionally, the study explored the impact of contrasting tillage practices and fertilizer nitrogen management options on maize yields. Using field data from 2019 and 2020, the DSSAT-CERES-Maize model was calibrated for plant growth stages, leaf area index-LAI, biomass, and yield. Data from 2021 were used to evaluate the model's performance. The treatments consisted of four nitrogen management options, viz., N0 (without nitrogen), N150 (150 kg N/ha through urea), GS (Green seeker-based urea application) and USG (urea super granules @150kg N/ha) in two contrasting tillage systems, i.e., CA-based zero tillage-ZT and conventional tillage-CT. The model accurately simulated maize cultivar’s anthesis and physiological maturity, with observed value falling within 5% of the model’s predictions range. LAI predictions by the model aligned well with measured values (RMSE 0.57 and nRMSE 10.33%), with a 14.6% prediction error at 60 days. The simulated grain yields generally matched with measured values (with prediction error ranging from 0 to 3%), except for plots without nitrogen application, where the model overestimated yields by 9–16%. The study also demonstrated the model's ability to accurately capture soil nitrate–N levels (RMSE 12.63 kg/ha and nRMSE 12.84%). The study concludes that the DSSAT-CERES-Maize model accurately assessed the impacts of tillage and nitrogen management practices on maize crop’s growth, yield, and soil nitrogen dynamics. By providing reliable simulations during the growing season, this modelling approach can facilitate better planning and more efficient resource management. Future research should focus on expanding the model's capabilities and improving its predictions further.

Efficient use of agricultural inputs, particularly fertilizers, is crucial for modern agriculture. Among the agricultural inputs, nitrogen (N) fertilizers play a crucial role in increasing the soil supply of N to crops, particularly cereal crops, which are highly responsive to N^1^. The use of nitrogen fertilizers in the production of food and fibre crops has grown significantly in recent decades, but the nitrogen use efficiency (NUE) remains low in cereal-based agro-ecosystems, with cereals typically taking up only 40–60% of applied nitrogen^2–4^. To maintain or increase grain yield, farmers often apply more N, leading to its excessive losses and resulting in negative environmental and socio-economic impacts such as groundwater contamination and greenhouse gas emissions^5,6^. Therefore, use of the slow-release N fertilizer source and urea deep placement (UDP) technology may be effective for reducing N losses and improving NUE in field crops. The UDP technology involves using large-sized fertilizer particles, known as urea supergranules or briquettes, which are placed at a depth of 7–10 cm near the root zone of the crop^7^. Previous studies have shown that these technologies can lead to higher crop yields and lower fertilizer use under conventional crop management conditions^8^ including the traditional intensive tillage etc., but for conservation agriculture (CA) based farming systems, the performance of these technologies need to be investigated.

In recent years the increasing demand for food and the need to reduce environmental impact has led to a growing interest in identifying economical and environmentally-friendly N management practices which increase the NUE while ensuring sustainable crop production^1^. Conservation agriculture, which includes three basic principle i.e., minimal soil disturbance, efficient residue cover/retention, and crop diversification^9,10^, has been shown to improve soil health and increased crop productivity. The past research evidences describe CA as a more sustainable form of agriculture in cereal-based cropping systems, in terms of resource use efficiency (water, fertilizers, and energy, etc.), profitability, soil quality, and agri-ecosystem resilience to climate change^11,12^. One potential alternative to diversify the traditional rice–wheat (RW) systems in North-West India is the integration of C_4_ crops like maize in this system^9,12^. The maize-wheat (MW) system is the third most important cropping system in the region and has the potential to expand in the face of emerging water crisis in the Indo-Gangetic Plains (IGP)^9,12^. Adopting CA-based practices in the MW system can provide dual benefits of superior food and fodder supply as well as improved soil health and crop productivity. The addition of crop residues has been shown to have improved soil quality by increasing soil organic carbon (SOC) content^13^, cation exchange capacity, aggregate stability^14^, and N mineralization^15,16^. However, the short-term immobilization of N by crop residues must be considered in the long-term strategy to enhance nutrient availability^17^. Therefore, determining the most appropriate way to manage N in these systems can be challenging due to the complexity of the soil N cycle and the influence of weather. One way to gain insight about these mechanisms is to use of simulation models, such as the Decision Support System for Agrotechnology Transfer (DSSAT). These models have the potential to provide valuable insights into N management in CA systems by simulating the effects of weather variables on soil N transformations, crop growth, and development^18^. Accurate simulation models are integral for resource management in agriculture, guiding judicious resource use by simulating growth and yield parameters. These models offer real-time decision-making insights, aiding in optimal choices for fertilizers, irrigation, and other inputs. Moreover, they facilitate comprehensive agricultural planning by considering soil conditions, weather variations, and input factors. Through this, simulation models contribute to a more efficient and sustainable farming approach, ensuring the judicious allocation of resources and enhancing productivity in harmony with environmental variables. Previous studies have evaluated the performance of the DSSAT-CSM and CENTURY-based soil module using data from cropping system experiments. For example, researchers have used these models to predict long-term trends in rice and wheat yields^19^, the effects of different N application rates on corn production^20^ and crop yields and N dynamics in a 50-year continuous maize production experiment^21^. These studies have shown that the DSSAT models can provide accurate predictions under a variety of conditions. In addition to their use in predicting crop yields, DSSAT models have also been used for yield forecasting^22–24^. These forecasts can be conducted prior to planting or during the growing season and can be used by farmers to manage expected crop production or by governments for agricultural planning^25^. However, it is important to note that the CERES-Maize model has not yet been well studied under contrasting tillage (CA vs CT) and N management options. Against this background, the present study was carried out with the following objectives: (1) To evaluate the CERES-Maize model for simulating the growth behavior of maize and soil nitrogen dynamics under emerging CA practices and application of novel fertilizer products such as urea supergranules (USG); (2) To assess the capabilities of DSSAT in evaluating the N application options and doses under different soil conditions (CA vs CT), and (3) To understand the effects of N management on crop growth and soil N dynamics under long-term CA-based maize-wheat system. This research will contribute to the ongoing effort to identify economical and environment-friendly nitrogen management practices that increases nutrient use efficiency (NUE) while ensuring sustainable crop production under CA-based systems.

## Results

### Temporal distribution of weather variables

**Figure 1:**
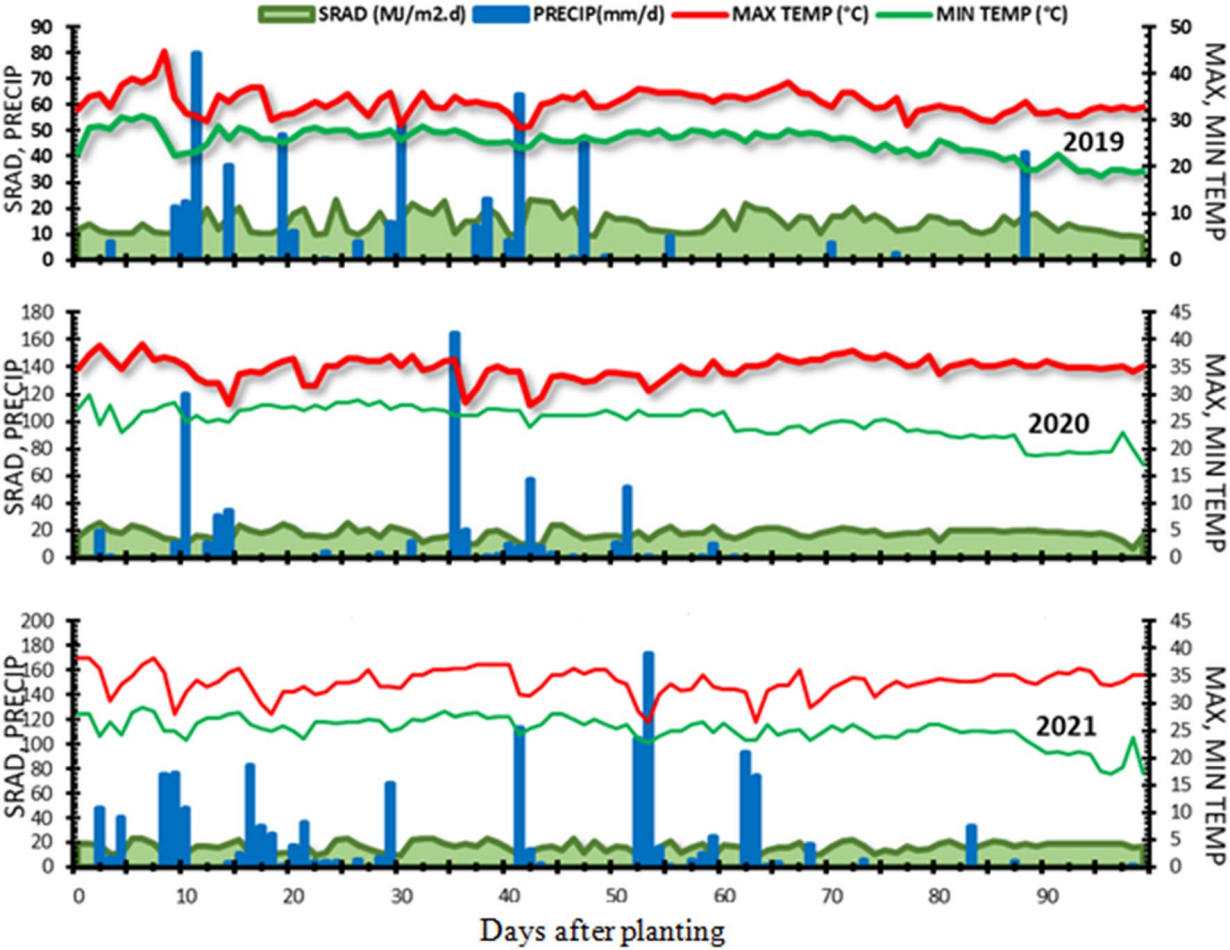
Weather conditions during experimentation (2019 to 2021). The abbreviations SARD, PRECIP, MAX TEMP, and MIN TEMP stand for solar radiation, precipitation, maximum temperature, and minimum temperature, respectively.

The amount of rainfall received during *kharif* 2019–2021 were 512.5, 587.5 and 1288.7 mm, respectively. The graphical representation of daily rainfall distribution, minimum and maximum temperature and solar radiation during simulation period have been presented in Fig. 1. The rainfall distribution during the experiment seasons *i.e.,* in 2019 (512.3 mm) and 2020 (587.5 mm) were almost comparable with distribution pattern almost similar, whereas 2021 (1288.7 mm) had higher rainfall, with higher amount of rainfall occurred during active growth stage of the cropping season.

### Model calibration

The six critical genetic coefficients for the model have been finally calibrated as presented in Table 1.

**Table 1:** Cultivar coefficients developed for maize variety PMH-1.

Parameters	DSSAT code	Value
Time period from emergence to the end of the juvenile phase (expressed in degree days above a base of 8 °C) during which the plant is not responsive to changes in photo period	P1	267.5
Coefficient of photoperiod sensitivity expressing extent to which development (expressed as days) is delayed for each hour increase in photo period above the longest photo period at which development proceeds at a maximum rate (which is considered to be 12.5 h)	P2	1.28
Degree days above a base of 8 °C from silking to physiological maturity	P5	900.8
Maximum possible number of kernels per plant	G2	550.5
The rate of potential kernel growth mg/day	G3	8.62
Degree days it takes for a leaf tip to emerge (phyllochron interval)	PHINT	50.0

**Figure 2:**
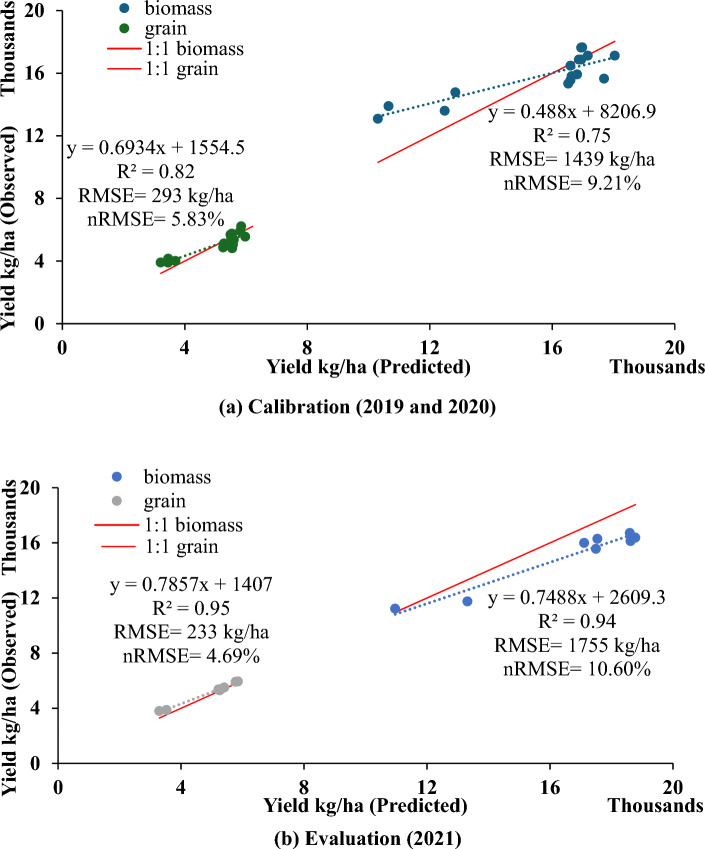
Observed vs. simulated grain and biomass yield of maize based on parameterization (**a**) and evaluation dataset (**b**).

**Figure 3:**
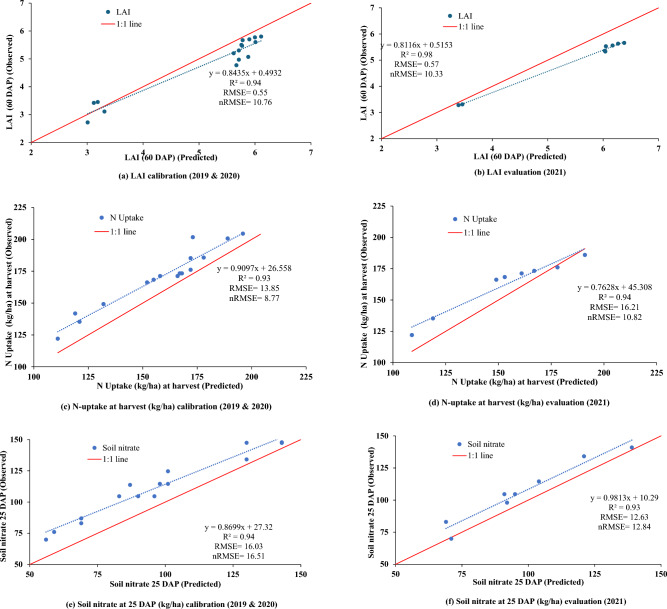
Observed vs. simulated phenological stages, LAI, (**a** and **b**), plant N-uptake (**c** and **d**) and soil NO_3_^−^-N (**e** and **f**) during 3 years of experimentation (2019–2021).

The performance statistics and goodness of fit are illustrated in Figs. 2a and 3a, c, and e. In the calibration years (2019 and 2020), the model showed strong performance with lower values of RMSE (293 and 1439 kg per hectare for grain and biomass, respectively) and nRMSE (5.83 and 9.21% for grain and biomass yield, respectively), indicating accurate simulation of crop yield. Similarly, for other parameters, RMSE and nRMSE values during the calibration years were approximately 0.56 and 10.76% for LAI, 13.85 and 8.77% for nitrogen uptake, and 16.03 and 16.51% for soil nitrate, respectively (Fig. 3a,c,e).

### Model evaluation

**Figure 4:**
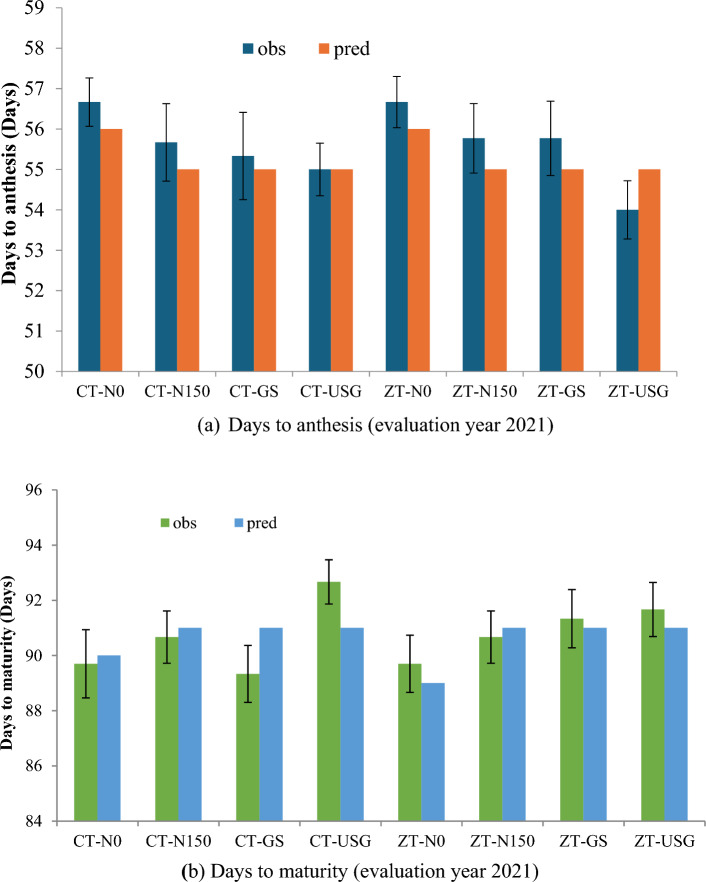
Observed vs. simulated phenological stages (**a**) days to anthesis, (**b**) days to maturity. Vertical bars for observed column represents standard error.

The genetic coefficients calibrated were used to simulate the yield and biomass of year 2021. The DSSAT-CERES-Maize model simulated maize cultivar anthesis, and physiological maturity at 54–57 and 90–92 DAP, respectively in all 3-years. (2019, 2020, 2021). The observed days to maturity and anthesis are given in Fig. 4 in which simulated days of the phenological stages lie within the 5% of those observed. Measured leaf area, nitrogen fluxes such as observations on nitrogen uptake and residual soil nitrate were also compared with those from simulation using DSSAT for all three years and statistical indicators suggest a good fit with acceptable RMSE and nRMSE (Fig.3b, d and f).

### Crop phenology

The results indicate that the model accurately predicted the time to anthesis of the crop, with an error of only 1–2 days (Fig. 4a), demonstrating good agreement between observed and simulated values (Fig. 4b). It's evident from the model that the timing of anthesis and physiological maturity is mostly determined by the variety of the crop, as treatments had minimal effect on these growth stages. However, under field conditions, slight variations were observed, particularly between treatments without nitrogen and those with nitrogen application. Treatments without nitrogen tended to delay anthesis but accelerated physiological maturity, supporting the idea that stress before flowering prolongs the period before anthesis but shortens it after. The simulated values for anthesis and physiological maturity closely matched the observed values, indicating the model's effectiveness. Treatments with nitrogen application required a longer time to reach physiological maturity, primarily due to the extended availability of nitrogen, which prolonged the post-anthesis duration of the crop.

### Leaf area index

The plant leaf area index (LAI) at any growth stage indicates the extent of the assimilatory system, which contributes to dry matter accumulation and partitioning. To validate the model using the LAI, we used data from the year 2021 tillage and nitrogen treatments in maize crop. Simulated LAI ranged from 3.0 to 6.0 across different treatments. During the first few days of the growing season, the simulation of LAI was nearly identical to the observed LAI for all the treatments except for the CT-N0 where model over-predicted the leaf area. At 60 days, the stage which corresponds to the crop’s maximum leaf area index, the model slightly under-predicted the LAI. Except for CT-N0, there were no significant differences in observed leaf area among treatments. The model accurately simulated the trend of LAI for all treatments, showing a rise in leaf area until grain formation, followed by a decrease as the plant transferred photosynthates to sink. The model closely simulated this trend for all treatments in 2021, with an RMSE of 0.57 and nRMSE of 10.3% (4b).

### Grain and biomass yield

**Figure 5:**
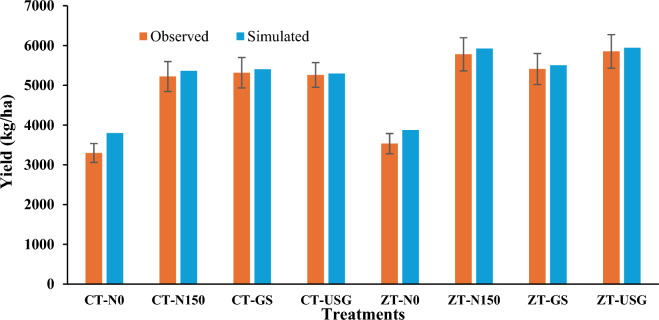
Comparison of observed versus simulated grain yield during evaluation year 2021. Vertical bars for observed column represents standard error.

In the evaluation year (2021), the root mean square error (RMSE) for grain and biomass yield was 233 and 1755 kg/ha, respectively (Fig. 2b). The nRMSE for measured and simulated grain yield and biomass yield were 4.69 and 10.60%, respectively. Maize yields varied significantly depending on tillage practices and fertilizer N levels (see Figs. 2b and 5). Generally, the simulated grain yields of maize closely matched the measured values across most treatments, with prediction errors ranging from 0 to 3 percent (Table 2). However, for N0 treatments in both conventional tillage (CT) and zero tillage (ZT), the model tended to over-predict grain yield, with prediction errors ranging from 9 to 16 percent (Table 2). Despite this discrepancy, the model performed well in predicting grain and biomass yields for the majority of treatments, demonstrating its effectiveness in simulating maize crop productivity.

**Table 2:** Prediction error of model (DSSAT) for grain yield in evaluation year (2021).

	Grain yield (kg/ha)		
Treatments	Observed	Simulated	Prediction error (%)
CT-N0	3297	3798	15.20
CT-N150	5221	5362	2.71
CT-GS	5316	5402	1.61
CT-USG	5260	5295	0.67
ZT-N0	3532	3873	9.65
ZT-N150	5778	5921	2.48
ZT-GS	5409	5503	1.74
ZT-USG	5852	5943	1.56

### Nitrogen dynamics and model’s sensitivity analysis

Both ammonium N and nitrate N are major forms of mineral N in soil. However, compared to ammonium N, nitrate N is generally available in principal form. In both the CT and ZT conditions, simulations showed that NH_4_^+^-N concentrations were similar and increased after adding fertilizer. However, it converted quickly to nitrate, and after a while, its concentration was very low (1–4 kg/ha). Model suggested that ammonia volatilization increased just after the fertilizer N application.

### Soil nitrate

**Figure 6:**
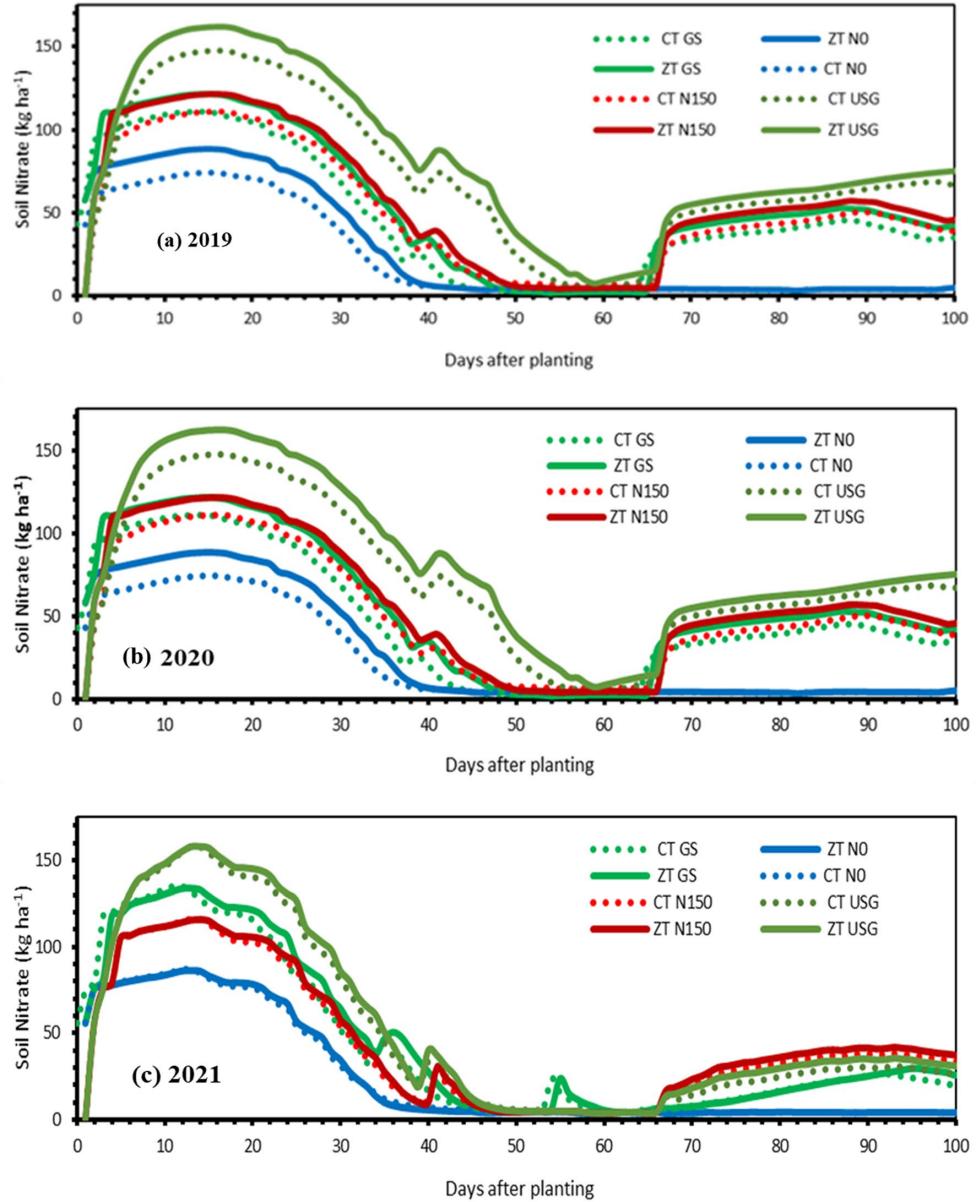
Simulated soil nitrate (NO_3_^−^-N) as influenced by different nitrogen management options under long-term contrasting tillage practices during 2019 (**a**), 2020 (**b**) and 2021 (**c**).

Unlike NH_4_^+^-N, not only nitrate–N rapidly diffused in soil but also steadily transported toward roots through mass flow via plant transpiration. The model accurately predicted soil nitrate levels, with RMSE and nRMSE values of 12.63 and 12.84%, respectively (Fig. 3f). The soil NO_3_^−^-N concentration varied significantly with fertilizer application and crop growth stages and constantly rose with fertilizer application. Consistently rising with nitrogen fertilization. Overall, nitrate levels increased notably with fertilizer use, especially in warmer conditions where nitrifying bacteria activity and nitrification rates are higher (Fig. 6). Warmer temperatures increased nitrate levels as they stimulate nitrifying bacteria and nitrification rates (Fig. 6). Generally, zero tillage (ZT) plots had more nitrate than conventional tillage (CT) plots. Among nitrogen management options, soil nitrate was highest in USG plots, followed by GS and 150N, while plots without nitrogen (N0) had the lowest levels. In 2021, all fertilizer application scenarios showed an extended period of nitrate stress.

### Nitrogen uptake

**Figure 7:**
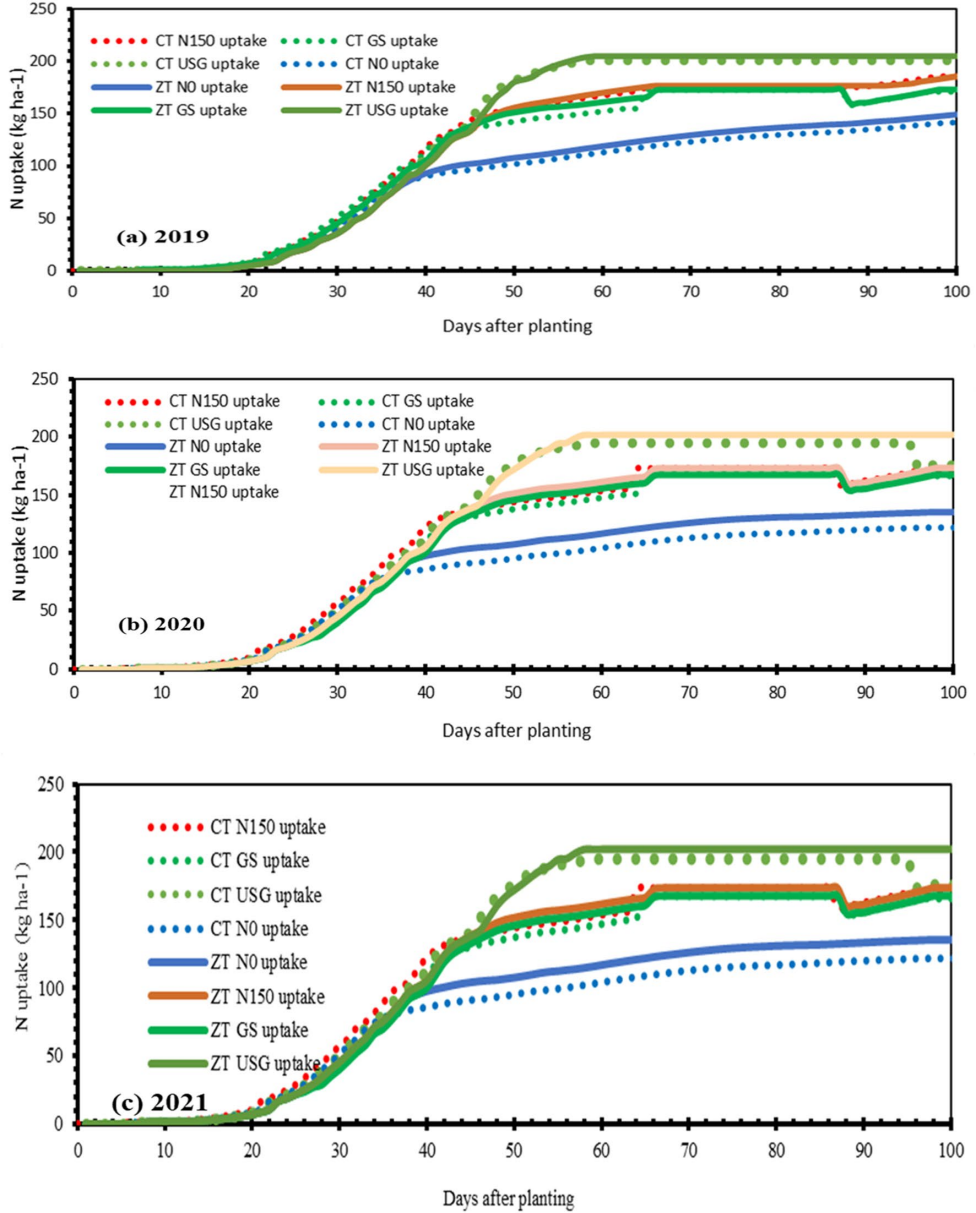
Simulated nitrogen uptake of maize as influenced by nitrogen management options under long-term contrasting tillage practices in 2019 (**a**), 2020 (**b**) and 2021 (**c**).

The observed and simulated nitrogen uptake showed a good match for evaluation year (2021), with an RMSE = 16.21 kg/ha and nRMSE = 10.82% (Fig. 3d). Nitrate–N concentration during active plant growth was low and highly dynamic, indicating rapid consumption matching supply rates. At 37 DAP and 65 DAP, despite fertilizer application, soil nitrate levels didn't accumulate much, as nitrate availability synchronized with peak crop growth and nitrogen demand (Fig. 6). The N uptake sharply increased after fertilizer application across all treatments. In this regard model’s prediction was in harmony with the findings of Jackson et al.^26,27^. It's also notable that higher nitrogen accumulation after basal nitrogen application was due to initial crop uptake absence. Nitrogen uptake patterns remained similar across three years, with maximum uptake in ZT-USG and lowest in CT-N0, despite higher rainfall in 2021 compared to 2019 and 2020 (Fig. 7). But clustered behavior was observed among nitrogen management options; for instance, CT-USG/ ZT-USG behaved similarly, as did CT-GS, CT-150N, ZT-GS, ZT-150N showed similar characteristics in the N uptake pattern. CT-N0 and ZT-N0 showed the similar N uptake capacity. However, on average ZT consistently outperformed CT across all N application options.

### Ammonia volatilization

**Figure 8:**
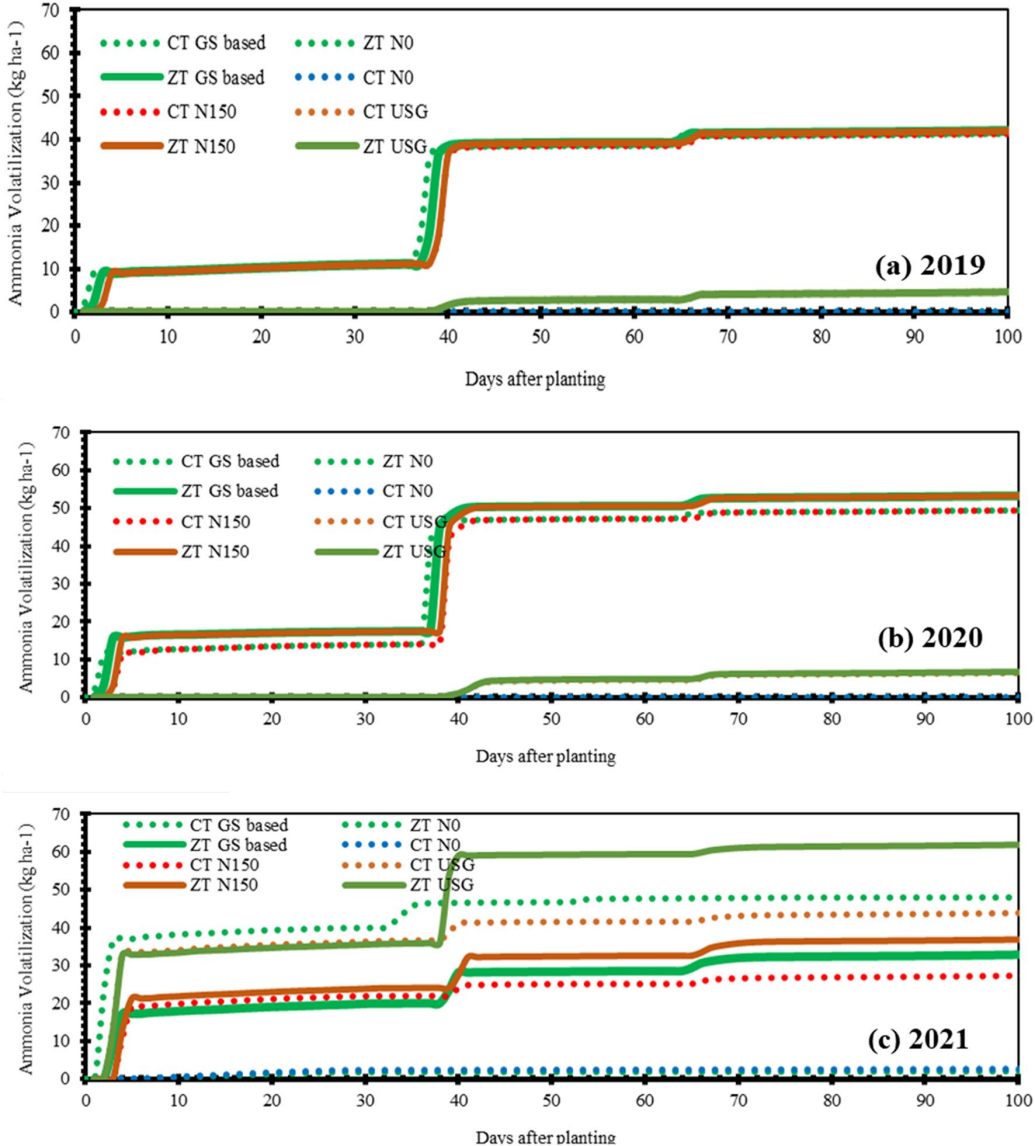
Simulated ammonia volatilization as influenced by different nitrogen management options under long-term contrasting tillage practices during 2019 (**a**), 2020 (**b**) and 2021 (**c**).

In this and following section, we only looked at simulated data and the discussion is based on comparing it with previous findings. The model showed that ammonia volatilization increased when NH_4_^+^-N levels rose after applying fertilizer, as NH_4_^+^-N acts as substrate for ammonia volatilization (Fig. 8). Ammonia volatilization depends on temperature and precipitation, increasing with higher temperatures. Heavy rainfall right after the basal nitrogen application in 2021 led to a big increase in volatilization across all N application options. However, the USG and zero tillage (ZT) reduced volatilization compared to conventional tillage (CT) and other N management options. USG fertilizer helped offset volatilization better in 2019 and 2020 compared to other nitrogen management options. The high temperatures in 2020 worsened ammonia loss through volatilization, especially with late heavy rainfall.

### Nitrate leaching

**Figure 9:**
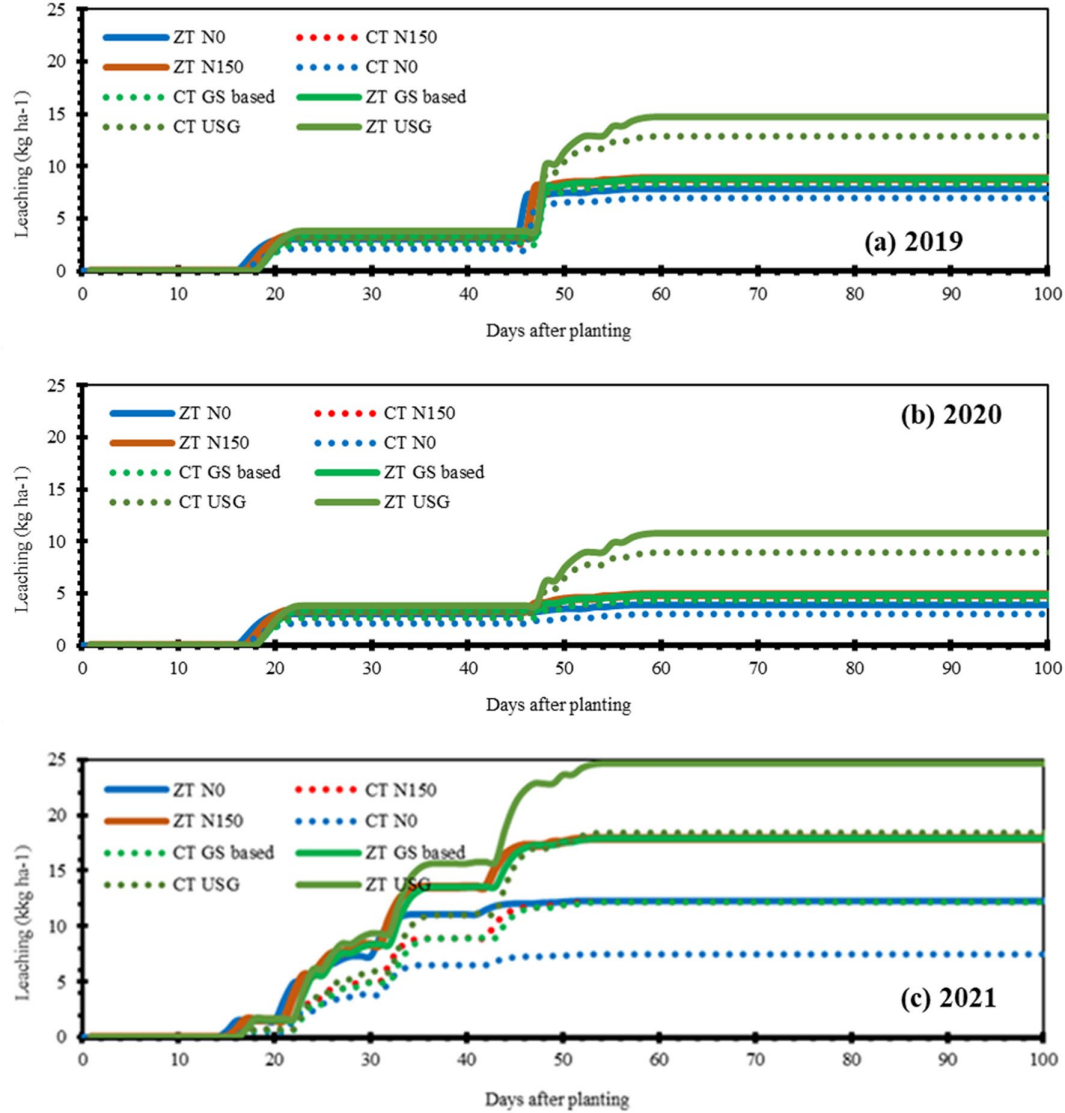
Simulated nitrate leaching as influenced by various nitrogen management options under long-term contrasting tillage practices during 2019 (**a**), 2020 (**b**) and 2021 (**c**).

Leaching in three crop seasons was mainly influenced by rainfall amount and its distribution throughout the cropping season. The model showed that leaching was highest in 2021, followed by 2019, and lowest in 2020. In 2019, consistent but not very heavy rainfall led to steady leaching, causing more leaching compared to 2020 (Fig. 9). However, in 2021, frequent rainfall events continuously wetted the soil, resulting in higher leaching rates.

Zero tillage (ZT) systems across all nitrogen application categories showed high nitrogen loss due to the soil's high porosity. High rainfall at the late stage of the crop triggered faster leaching. The peak NO_3_^−^-N leaching coincided with precipitation and a fair conclusion can be drawn that rainfall triggers leaching and loss of nitrate into the deeper soil layers depended on water flux during each drainage event as stated by Godwin and Jones^28^. However, considering grain yield, nitrate leaching didn't seem to have much effect. The reason lies in the fact that leached nitrate can rise again with the capillary action to the root zone as it is water soluble, and model also takes into account this fact considering the water flux going upward with capillary action^29^. The information is important for improving our understanding of the crop–soil N dynamic processes.

## Discussion

The distinct behavior of soil and crop residue decomposition in conventional and conservation agriculture (CA) significantly impact crop growth and nitrogen dynamics. The successful understanding of these complex dynamics and underlying processes has been achieved through the effective utilization of simulation models^30–32^.

### Phenology

Understanding the different growth stages of maize, like when it flowers and when it's fully grown, is really important for how much corn we can harvest^33^. In this study, the DSSAT-CERES-Maize model successfully simulated the phenological stages of maize cultivars, showing good agreement with observed values. The model also demonstrated the impact of nitrogen treatments on anthesis and physiological maturity, with no nitrogen delaying anthesis but advancing physiological maturity (Fig. 4). Additionally, the model effectively simulated the leaf area index (LAI) and grain yield (Figs. 3a, b and 5). This effectiveness of the model was also observed by Chisanga et al.^34^, DeJonge et al.^35^ and Timsina and Humphreys^19^. In our study, the LAI simulation closely matched the observed values, indicating the model's ability to capture the assimilatory system's contribution to dry matter accumulation and partitioning^18^.

### Grain yield

Although the model slightly over-predicted grain yield in treatments without nitrogen (N0), it generally matched the measured values well, particularly in treatments with fertilizer nitrogen application. Similar DSSAT simulation findings were reported by Timsina and Humphreys^18^, Liu et al.^21^ and Yang et al.^36^ which showed a poor response of DSSAT for N0 treatment than the treatments with fertilizer N and they attributed this to the higher sensitivity of model to the fertilizer N application. Jones and Kiniry^37^ also demonstrated the sensitivity of the DSSAT model to the accurate specification of initial mineral N to the simulation response to yield and found to have reported a similar outcome. If initial mineral N is not measured accurately, then the reliability of the simulated response to N must be questioned. Thus, the DSSAT model demonstrated “good to excellent” agreements in simulating the maize yield for the treatments with fertilizer N application. In all the ZT treatments, increase in the grain yield was fairly captured by the model and it was primarily due to higher nitrogen availability and uptake due to more favourable soil conditions in terms of higher organic carbon content, lower bulk density, high water retention and moderation of the soil temperature^38^.

### Nitrogen dynamics

Ammonium and nitrate are the two major forms of mineral nitrogen in soil, with nitrate being the more readily available form. Ammonium is quickly nitrified to nitrate upon addition to the soil. The diffusion rate of nitrate and ammonium in the rhizosphere differs significantly, with ammonium being adsorbed to the soil solid phase and having a much lower effective diffusion coefficient than nitrate^39^. Our model simulations showed that NH_4_^+^-N concentrations increased following fertilizer application but quickly nitrified into nitrate, becoming negligible. Previous studies have shown that soil ammonium N has no correlation with wheat yield^40,41^. Consistent with experimental results of Li et al.^42^ and Zhao et al.^43^, our simulations revealed higher nitrate content in zero tillage plots compared to conventional tillage plots, attributed largely to initial nitrate content and favorable nitrification conditions. Nitrate content was highest in plots with USG nitrogen management and lowest in N0 plots. Despite excessive leaching and volatilization in 2021 causing extended period of nitrate stress, the USG nitrogen application helped alleviate this stress. Nitrate accumulation in maize had multiple benefits, including ensuring smooth & consistent nitrogen nutrition, promoting vigorous growth, and increasing yield. Our DSSAT model provided dynamic soil nitrogen data and indicated a minor influence of nitrogen dose on native nitrogen release and leaching loss of nitrate. The model's ability to simulate NO_3_^−^-N dynamics and estimate leaching loss and soil N balance under various management practices and weather conditions improves our understanding of crop-soil nitrogen dynamics. Further, our simulations showed that the ammonia volatilization and aboveground biomass nitrogen uptake were 21 and 67 percent of the total nitrogen available to the crops (applied fertilizer nitrogen and mineralized nitrogen). Assimilation of nitrate in plant tissues, especially when soil nitrate levels are high, is common across all plants and doesn’t show usually any detrimental effect^44,45^. In fact, nitrate serves as a safe nitrogen source, unlike ammonium, which showed inhibited/poor vegetable growth^44,45^. Such high accumulation of nitrate–N in maize can offer multiple beneficial effects. Firstly, it ensures a smooth N nutrition and when the external N falls short of supply, the nitrate stored in vacuoles can be released to cytoplasm for plant, contributing to prolonged growth periods and increased photosynthesis ^46,47^. Secondly, a high accumulation of nitrate–N in plants promotes vigorous growth. With continuous growth, the stored nitrate–N decreases significantly, and at flowering stage, plants with high nitrate–N accumulation still contain nitrate, while those with low nitrate accumulation have nearly all nitrate utilized. Consequently, plants with higher nitrate–N accumulation have a longer growth period and can produce more photosynthetic products. This likely explains why maize plants with higher soil nitrate and fertilizer application took longer to reach physiological maturity compared to those with lower soil nitrate and less/no fertilizer application (Fig. 3). Thus, nitrate accumulation in plants ensures abundant growth and higher yields^46,47^.

## Conclusions

Financial and time constraints coupled with drudgery in field experiments can be alleviated with use of crop models such as DSSAT in field crop investigations. The goal of using DSSAT is simulation of crop growth and nutrient dynamics in a wide range of environmental conditions. In our study, we calibrated the genetic coefficients of a maize cultivar (PMH1) using CSM-CERES-Maize model and demonstrated how its calibrations brought about a close simulation of crop growth and dynamic processes involving the fate of nitrogen. The Model satisfactorily simulated grain yield (RMSE = 233 kg/ha) and biomass yield (RMSE = 1755 kg/ha). The simulated LAI at 60 DAP was in accordance with the measured leaf area index, with an nRMSE of 10.3%. Simulated maximum LAI ranged from 3.0 to 6.0 across different treatments. All nitrogen levels in ZT treatments recorded higher LAI and grain yield than CT treatments. The model also successfully simulated different nitrogen levels in zero tillage (ZT) and conventional tillage (CT) plots, demonstrating higher LAI and grain yield in ZT treatments. It effectively simulated nitrogen dynamics, including inorganic nitrogen application, native nitrogen mineralization, crop nitrogen uptake, nitrate leaching, and ammonia volatilization losses under varying nitrogen levels. Given the importance of maize and the cultivar used in our study, generated genetic coefficients of cultivar in combination with the validated model will help growers/producers and researchers in making site specific and real time decisions. Additionally, it can also help understand complex interactions between genotype, environment, and management (G × E × M) and predict optimal tillage and nitrogen strategies for maximizing maize yield under different climatic conditions, facilitating the timely transfer of agricultural technology.

## Methods

### Description of experiment site

The experimental data was collected from maize in a long-term ongoing conservation agriculture-based maize-wheat system experiment (started since *kharif* 2012) for a period from 2019 to 2021. The field site (block ‘9B’) is located in the research farm premises of ICAR-Indian Agricultural Research Institute (IARI), New Delhi (28° 40′N latitude, 77° 11′E longitude and an altitude of 228 m above sea level), representing Indian Trans-Gangetic Plains Zone (Agro Climatic Zone-VI) with a sub-tropical and semi-arid climate. The average annual rainfall of the site is 735 mm with hot dry summer, wet monsoon and cold winters. The site receives about 80% of its total rainfall during the monsoon season (July–September). At the start of the experiment, physico-chemical soil properties were determined using standard procedure as outlined by Parihar et al.^11^. The detailed initial soil properties are as presented in Supplementary Table 1.

### Description of imposed treatments and adopted agrotechniques

In this study, we investigated the growth behavior of maize crops and soil nitrogen dynamics under different tillage and nitrogen management practices. The experiment involved a split plot design with three replications. We compared four different nitrogen sources/method and nitrogen rates in sub plots viz., N0 (no nitrogen), N150 (150 kg N/ha through urea), GS (Green seeker-based urea application) and USG (urea super granules @150kg N/ha). These were tested in two different tillage methods in main plots: CA based zero tillage (ZT) and conventional tillage (CT). The resulting eight treatments are described in detail in Table 3.

**Table 3:** Description of imposed treatments in the experiment during 2019–2021.

S. no	Tillage and residue management	Nitrogen management	Notations
1	Conventional tillage with residue incorporation (CT)	Control (without-N application) (N0)	CT-N0
2	Conventional tillage with residue incorporation (CT)	Recommended dose of N @ 150 kg N/ha applied through urea (N150)	CT-N150
3	Conventional tillage with residue incorporation (CT)	1/3rd of recommended dose + Green Seeker-GS based application of split applied N (GS)	CT-GS
4	Conventional tillage with residue incorporation (CT)	50% of recommended dose of N applied as basal through urea super granules + GS based application of split applied N (USG)	CT-USG
5	Zero tillage flat bed with residue retention (ZT)	Control (without-N application) (N0)	ZT-N0
6	Zero tillage flat bed with residue retention (ZT)	Recommended dose of N @ 150 kg N/ha applied through urea (N150)	ZT-N150
7	Zero tillage flat bed with residue retention (ZT)	1/3rd of recommended dose + Green Seeker-GS based application of split applied N (GS)	ZT-GS
8	Zero tillage flat bed with residue retention (ZT)	50% of recommended dose of N applied as basal through urea super granules + GS based application of split applied N (USG)	ZT-USG

In all CT plots, 2.91–2.94 ton/ha crop residue of preceding crop was incorporated into the soil during the study years (2019–2021). Conversely, in the ZT plots, the preceding crop residue was retained on the soil surface. ZT plots were prepared in June 2012 and remained undisturbed throughout the years. During the experimentation period (2019–2021), maize variety PMH1 was manually sown in the first week of July during the rainy season and was harvested in the second week of October. A recommended dose of 60 kg P_2_O_5_, 40 kg K_2_O/ha was applied as basal directly at the time of seeding. The control plot (without N applied plot) received only P_2_O_5_ and K_2_O, while the recommended dose of N (RDN) plots received 150 kg N/ha with 50 kg/ha as a basal dose and the remaining N was split into two equal portions and point-placed at 7–10 cm depth near the root zone at 36 days after planting (DAP) and 61 DAP. Irrigation was applied at critical stages according to the crop water requirement. The water application and measurement procedures followed those outlined by Parihar et al.^48^.

### Measurement of leaf area index, crop yields and plant N uptake

In the present study, crop yields and nitrogen dynamics were measured to evaluate the impact of different tillage and nitrogen management practices. Leaf area was measured periodically at 25 days interval using a leaf area meter (Model LI-COR-3100). In this paper, LAI at 60 DAP is discussed which corresponds to the stage at which the crop attains its maximum LAI values. The leaf area index (LAI) was calculated as follows:

1 $${\text{ Leaf}}\;{\text{area}}\;{\text{index }}\left( {{\text{LAI}}} \right){ } = \frac{{{\text{Total}}\;{\text{leaf}}\;{\text{area }}^{{}} }}{{{\text{Ground}}\;{\text{area}}\;{\text{accupied}}}}$$

Biomass and yields of the maize crop were recorded at harvest as per the standard protocol^11^. Crop harvesting was done manually, and maize grain samples were collected at harvest, oven-dried at 65–70 °C for 48 h and weighed. The nitrogen (N) content in both grain and stover samples was determined using a CHNS analyzer, which employs the “Dumas method” involving dry, rapid and complete sample combustion. N uptake was then calculated using the formula:

2 $$ {\text{N}}\;{\text{uptake}}\;{\text{(kg/ha)}}\;{\text{in}}\;{\text{grain/stover = }}\left[ {{\text{\% N}}\;{\text{in}}\;{\text{grain/stover}} \times {\text{grain/stover}}\;{\text{yield}}\left( {{\text{kg}}/{\text{ha}}} \right)} \right] $$

The total N uptake (kg/ha) was calculated as the sum of N uptake in grain and N uptake in stover using equation as follows:

3 $${\text{Total}}\;{\text{N}}\;{\text{uptake}}\;\left( {{\text{kg}}/{\text{ha}}} \right) = {\text{N}}\;{\text{uptake}}\;{\text{in}}\;{\text{grain}}\;\left( {{\text{kg}}/{\text{ha}}} \right) + {\text{N}}\;{\text{uptake}}\;{\text{in}}\;{\text{stover}}\;\left( {{\text{kg}}/{\text{ha}}} \right)$$

### Determination of mineral-N (NH_4_^+^-N and NO_3_^−^-N)

Soil samples were collected randomly at 25 days interval from three places in each plot at six different depths (0–15, 15–30, 30–45, 45–60, 60–75, and 75–100 cm) using a soil sampler with a 5 cm internal diameter. The samples were then composited for each plot and mineral-N fractions were determined using standard methods^49–51^. To determine the mineral-N (NH_4_^+^ and NO_3_^–^-N) content, moist soil samples were extracted with a 2M KCl solution (soil:solution ratio of 1:5) by shaking for 1 h using a mechanical shaker^50,51^. Soil mineral-N content was estimated using steam distillation methods^50^, first with MgO for NH_4_^+^-N and then with MgO and Devarda’s alloy separately for NO_3_^–^-N reduction. Ammonium in the extract was estimated from the NH_3_ liberated with distillation of the extract with MgO, creating the necessary alkaline condition for ammonia formation. In the same extract, a reducing agent (Devarda’s alloy) was added to reduce the NO_3_^−^-N to ammonia. The mineral-N content of soil samples was expressed on a dry weight basis. While the soil nitrate was assessed periodically, this paper’s primary emphasis lies in the examination of both observed and simulated soil nitrate levels at the 25 DAP. This stage is used for simulation because at this stage nitrate uptake by the crop is rapid, which presents a challenging scenario for the model to accurately replicate the soil nitrate dynamics.

### Crop growth simulation using CERES-Maize

#### Model description

**Figure 10:**
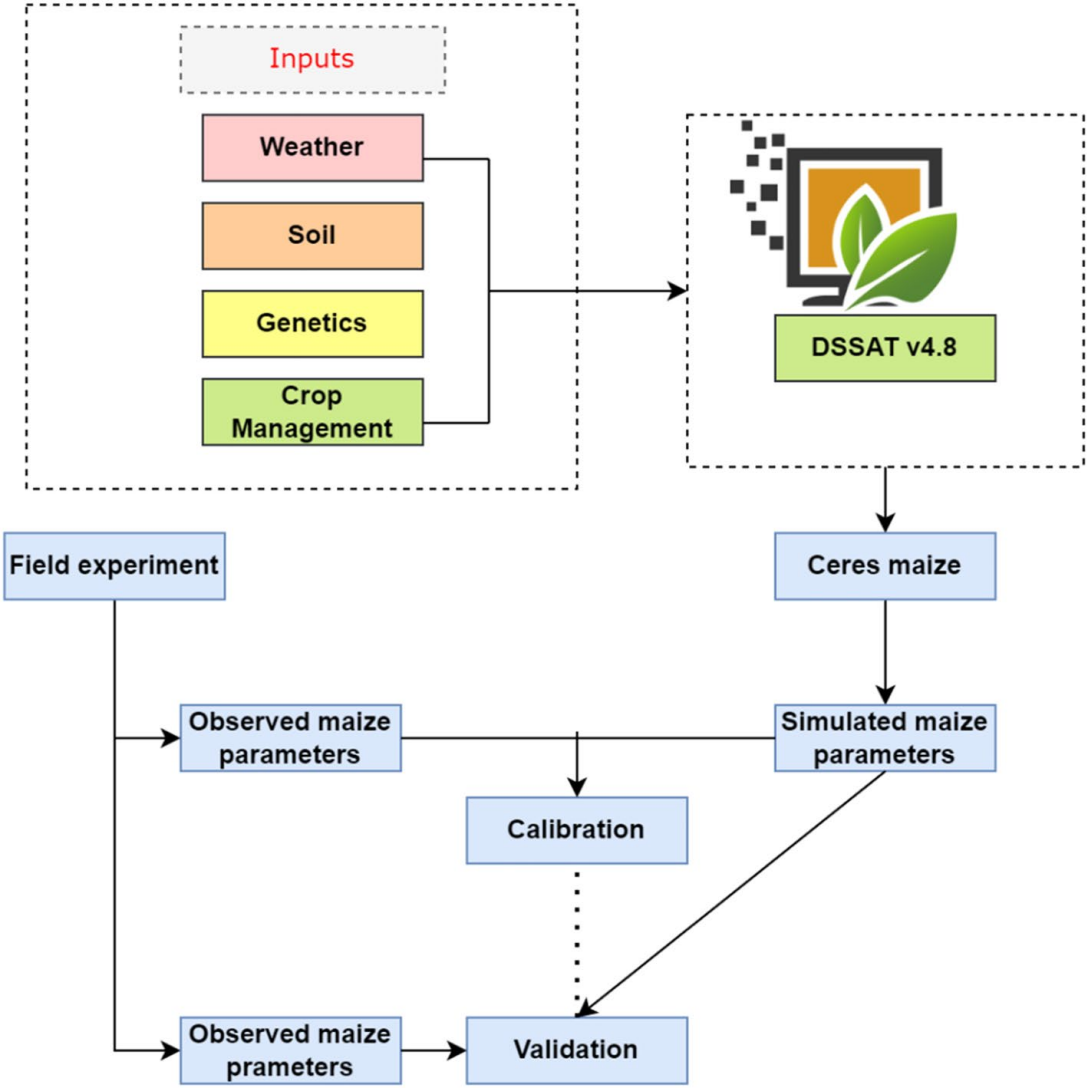
Flowchart illustrating the functioning of the DSSAT CERES-Maize model.

In this study, simulations were conducted using the CERES-Maize model, which is incorporated within the input modules of the Decision Support System for Agro-technology Transfer (DSSAT) model version 4.8, with a CENTURY soil organic matter module for modeling complex decomposition dynamics of soil organic matter and surface residue layer. A detailed description of the CSM-CERES-Maize model can be found in Ritchie et al.^52^. The CERES-Maize model is a dynamic mechanistic model that simulates the phenological development and growth of maize on a daily time step in response to environmental factors, such as soil and climate, and management factors, such as crop variety, fertilization, planting conditions, and irrigation (Fig. 10). The model has been extensively tested for different soil types, climatic conditions, and maize hybrids^53,54^. Crop growth in the CSM-CERES-Maize model is controlled by phenologically defined growth stages driven by energy input in the form of growing degree-days (GDD)^55^. The CERES-Maize model uses growing degree days (GDD) to calculate the developmental stage of the crop, with GDD being a measure of heat accumulation during the growing season. The model also simulates the uptake of nitrogen by the crop, taking into account factors such as soil properties, weather conditions, and management practices^55^. The DSSAT model is a collection of independent programs, including crop, weather, soil, and water modules, which are closely coupled with the cropping system model as the core, simplifying the simulation of crop rotations. The model simulates crop growth and development from planting to maturity on a time step based on physiological processes describing the response of a crop to soil and aerial environmental conditions. Potential growth is dependent on photosynthetically active radiation and its interception, whereas actual biomass production on any day is constrained by suboptimal temperatures, soil water deficits, and nitrogen deficiencies. The input data required for the DSSAT models include daily weather data, soil characterization data, cultivar coefficients, and crop management information (Fig. 10). The soil water balance is determined on a daily basis as a function of precipitation, irrigation, transpiration, soil evaporation, runoff, and drainage from the bottom of the profile.

#### Model calibration and evaluation

To ensure accurate simulations in DSSAT, cultivar coefficients tailored to local conditions and crop varieties must be calibrated under optimum conditions^21,59^. Six cultivar coefficients (P1, P2, P5, G2, G3, and PHINT)^55^, three representing crop duration (P1, P2 and P5), two representing grain filling (i.e., G2 and G3), and one representing leaf area and leaf area duration by phylochron interval between successive leaf tip appearances (PHINT), were used in the CSM-CERES-Maize model, and these were calibrated for the PMH1 cultivar using the DSSAT-GLUE tool to match simulated and measured biometric variables and phenological stages. When the day length is less than 14 h, the silking rate is governed by P1 as the second variable P2 has no role in governing the phenology of the maize. P2 is used when the day length is more than 14 h. The P1 was adjusted till the simulated value matched with observed value. Similarly, P5 was adjusted to calibrate the duration between silking to physiological maturity. In a nut shell, first three variables (P1, P2 and P5) govern the crop duration; the other two (G2 and G3) control the grain yield and the last one (PHINT) controls the leaf area and leaf area duration.

For calibration, average values of key crop parameter such as days to 50% anthesis, and physiological maturity, maximum LAI, soil nitrate content, nitrogen uptake and, grain and biomass yield was obtained from nitrogen and water stress free plots in the 2019 and 2020 growing seasons. Subsequently, the calibrated coefficients' performance was validated against an independent dataset from a 2021 field experiment exploring tillage and nitrogen interactions, utilizing the same parameters as in calibration.

#### Model performance evaluation

Comprehensive evaluation of the DSSAT model performance was carried out by comparing the observed and model-simulated data using deviation statistics, such as prediction error (PE), root mean square error (RMSE), normalized RMSE (nRMSE), and coefficient of determination (R^2^). Additionally, regression analysis was performed to compare the simulated and measured grain yields. The prediction error, PE is defined as follows:

4 $${\text{PE}} = \frac{{\left( {P_{i} - O_{i} } \right)}}{{O_{i} }} \times 100$$

where P_i_ is predicted/simulated value, O_i_ is observed value.

Prediction is considered to be excellent if PE value is close to zero.

The root mean square error (RMSE)^60^ was used to calculate the fitness between the simulated and measured results. The RMSE summarizes the average difference between observed and predicted values and is expressed as follows:

5 $${\text{RMSE}}=\sqrt{\frac{1}{n}\sum_{i=1}^{n}{\left({P}_{i}-{O}_{i}\right)}^{2}}$$

The normalized RMSE is expressed as RMSE as percent over the mean observed value.

6 $${\text{nRMSE}}\;\left( \% \right) = \left( {{\text{RMSE}}/{\overline{\text{O}}}} \right) \times {1}00$$

where P_i_ is predicted/simulated value, O_i_ is observed value, Ō is observed mean and n is the number of samples. The nRMSE (%) shows the relative difference between the predicted/simulated and observed data. The prediction is considered excellent, good, fair and poor for the nRMSE < 10%, 10–20%, 20–30% and > 30%, respectively^60,61^.

### Permit statement

This study was conducted at IARI, and the authorship and acknowledgements have been appropriately attributed to each contributor based on their respective contributions.

The Experimental research and field studies on maize plants (cultivated), including the collection of plant material, were complied with relevant institutional, national, and international guidelines and legislation.

## Supplementary Information

Supplementary Information.

## Data Availability

The datasets used and/or analyzed during the current study available from the corresponding author on reasonable request.

## Abbreviations

ANOVA: Analysis of variance
CA: Conservation agriculture
CERES: Crop Environment Resource Synthesis
CSM: Cropping system model
CT: Conventional tillage
DAP: Days after planting
DAS: Days after sowing
Fig.: Figure
GDD: Growing degree days
GS: Green seeker-based urea application
/ha: Per hectare
IGP: Indo-Gangetic Plains
LAI: Leaf area index
nRMSE: Normalized root mean square error
PE: Prediction error
PHINT: Phyllochron interval
R^2^: Coefficient of determination
RMSE: Root mean square error
SOM: Soil organic carbon
UDP: Urea deep placement
USG: Urea super granules
ZT: Zero tillage
