# Supplementary material for: Modeling maize growth and nitrogen dynamics using CERES-Maize (DSSAT) under diverse nitrogen management options in a conservation agriculture-based maize-wheat system
Source: Sci Rep. 2024 May 23;14:11743. doi: 10.1038/s41598-024-61976-6 (PMC11639734; doi:10.1038/s41598-024-61976-6)
Supplement: Supplementary file 1 — Supplementary Information. [file 41598_2024_61976_MOESM1_ESM.docx]

**Supplementary Table 1:** Physical and chemical soil properties prior to the experimental onset in 2019.

| **Parameters** | **Soil profile depth (cm) CT plots** | | | | | | **Soil profile depth (cm) ZT plots** | | | | | |
| --- | --- | --- | --- | --- | --- | --- | --- | --- | --- | --- | --- | --- |
|  | **0-15** | **15-30** | **30-45** | **45-60** | **60-75** | **75-100** | **0-15** | **15-30** | **30-45** | **45-60** | **60-75** | **75-100** |
| Lower limit (cm^3^/cm^3^) | 0.09 | 0.08 | 0.09 | 0.09 | 0.09 | 0.09 | 0.08 | 0.07 | 0.07 | 0.07 | 0.07 | 0.07 |
| Upper limit, drained (cm^3^/cm^3^) | 0.26 | 0.26 | 0.26 | 0.26 | 0.26 | 0.26 | 0.27 | 0.26 | 0.26 | 0.26 | 0.26 | 0.26 |
| Saturated water content | 0.42 | 0.41 | 0.39 | 0.39 | 0.39 | 0.35 | 0.44 | 0.42 | 0.41 | 0.39 | 0.39 | 0.35 |
| Root growth factor (0–1) | 1.00 | 0.08 | 0.07 | 0.06 | 0.50 | 0.30 | 1.00 | 0.08 | 0.07 | 0.06 | 0.50 | 0.30 |
| Saturated Hydraulic conductivity (cm/h) | 1.0 | 0.9 | 0.9 | 0.8 | 0.8 | 0.7 | 1.00 | 0.90 | 0.90 | 0.80 | 0.80 | 0.70 |
| Bulk density (g/cm^3^) | 1.48 | 1.45 | 1.47 | 1.47 | 1.47 | 1.47 | 1.39 | 1.43 | 1.41 | 1.40 | 1.41 | 1.41 |
| Organic carbon (%) | 0.43 | 0.42 | 0.33 | 0.31 | 0.24 | 0.21 | 0.65 | 0.56 | 0.47 | 0.39 | 0.28 | 0.19 |
| Stable organic carbon (%) | 0.19 | 0.17 | 0.14 | 0.13 | 0.10 | 0.08 | 0.28 | 0.24 | 0.19 | 0.15 | 0.12 | 0.08 |
| NH_4_^+^-N (ppm) | 4.11 | 1.43 | 3.2 | 0.93 | 0.32 | 0.38 | 4.32 | 2.12 | 1.33 | 0.93 | 0.81 | 1.12 |
| NO_3_^—^N (ppm) | 9.32 | 7.65 | 5.60 | 4.17 | 3.19 | 2.12 | 10.87 | 8.61 | 6.15 | 3.39 | 2.28 | 1.98 |
| Clay (< 0.002 mm) (%) | 25 | 25 | 25 | 24 | 24 | 24 | 25 | 25 | 25 | 24 | 24 | 24 |
| Silt (0.05 – 0.002) (%) | 15 | 15 | 15 | 16 | 16 | 16 | 15 | 15 | 15 | 16 | 16 | 16 |
| pH in water | 7.3 | 7.3 | 7.3 | 7.3 | 7.4 | 7.4 | 7.3 | 7.3 | 7.3 | 7.3 | 7.4 | 7.4 |
